# Cardiac Remodeling in Chronic Kidney Disease

**DOI:** 10.3390/toxins12030161

**Published:** 2020-03-05

**Authors:** Nadine Kaesler, Anne Babler, Jürgen Floege, Rafael Kramann

**Affiliations:** 1 Clinic for Renal and Hypertensive Disorders, Rheumatological and Immunological Disease, University Hospital of the RWTH Aachen, 52074 Aachen, Germany; 2 Department of Internal Medicine, Nephrology and Transplantation, Erasmus Medical Center, 3015 GD Rotterdam, The Netherlands

**Keywords:** uremia, uremic cardiomyopathy, organ crosstalk, cardiorenal syndrome, chronic kidney disease, left-ventricular hypertrophy, heart failure, cardiac fibrosis

## Abstract

Cardiac remodeling occurs frequently in chronic kidney disease patients and affects quality of life and survival. Current treatment options are highly inadequate. As kidney function declines, numerous metabolic pathways are disturbed. Kidney and heart functions are highly connected by organ crosstalk. Among others, altered volume and pressure status, ischemia, accelerated atherosclerosis and arteriosclerosis, disturbed mineral metabolism, renal anemia, activation of the renin-angiotensin system, uremic toxins, oxidative stress and upregulation of cytokines stress the sensitive interplay between different cardiac cell types. The fatal consequences are left-ventricular hypertrophy, fibrosis and capillary rarefaction, which lead to systolic and/or diastolic left-ventricular failure. Furthermore, fibrosis triggers electric instability and sudden cardiac death. This review focuses on established and potential pathophysiological cardiorenal crosstalk mechanisms that drive uremia-induced senescence and disease progression, including potential known targets and animal models that might help us to better understand the disease and to identify novel therapeutics.

## 1. Chronic Kidney Disease

Chronic kidney disease (CKD) affects an increasing number of patients worldwide and is associated with dramatically increased morbidity and mortality [1,2]. Recent data suggest that CKD currently affects more than 10% of the population in the developed world [3,4]. 

Diabetes mellitus and high blood pressure are among the most prevalent risk factors for the development of CKD and are responsible for the majority of cases. Other conditions that affect the kidneys are glomerulonephritis, the third most common type of kidney disease, inherited diseases, such as polycystic kidney disease, and loss of renal tissue due to infections, malformations or urinary tract obstruction. Repeated episodes of acute kidney injury and certain therapeutics, such as non-steroidal anti-inflammatory drugs (NSAIDs) can also contribute to CKD [2].

In end-stage renal failure, dialysis and subsequent kidney transplantation are the only available treatment options apart from palliative care. However, not all patients qualify for kidney transplantation and transplant waiting times are often long. Thus, patients usually undergo many years of dialysis treatment. Longstanding uremia in turn promotes cardiovascular disease. Consequently, the leading causes of death in dialysis patients are sudden cardiac death and recurrent heart failure due to cardiac and vascular remodeling [5]. 

### Cellular Crosstalk in the Heart

The mammalian heart is a highly interactive complex of cardiac muscle cells, extracellular matrix (ECM) and vessels. Other essential cell types include endothelial cells, fibroblasts, vascular smooth muscle cells and perivascular cells [6,7]. Studies, using state-of-the-art methods such as single-cell transcriptomics, suggest that all cardiac cell types communicate vigorously with one another in homeostasis and disease [8,9,10]. Each cardiomyocyte is in physical contact with at least one capillary, allowing mechanical and paracrine crosstalk between at least four key cell types, namely, cardiomyocytes, endothelial cells, vascular smooth muscle cells (VSMCs) and pericytes/fibroblasts [11]. Cardiomyocytes crosstalk with endothelial cells and fibroblasts by secreting various specific growth factors [7]. Various lines of evidence suggest that endothelial cells crosstalk with cardiomyocytes and are key players in angiogenesis and vasomotor tone control by secreting angiocrine factors such as nitric oxide or endothelin-1 [12]. One example of paracrine intercellular crosstalk inside the myocardium is vasomotion. Endothelial cells are directly exposed to shear stress, contrary to VSMCs. To facilitate vasomotion, endothelial cells release nitric oxide in response to shear stress, thus signaling to the VSMCs to dilate. The cardiac morphology and function can be affected by further external and internal stimuli. 

## 2. Pathology and Pathophysiology of the Cardiorenal Syndrome

Kidney and cardiac health are highly linked to each other, with diseases of either organ affecting the other organ. In the following, we aim to give an overview of the mechanisms and relevant factors that have been reported to be involved in cardiac remodeling due to kidney injury, i.e., cardiorenal syndrome.

The presence of CKD and end-stage renal disease (ESRD) leads to cardiac remodeling with hypertrophy, fibrosis and capillary loss [13]. Uremic cardiomyopathy affects about 80% of hemodialysis patients [14] and is the main cause of death in this cohort. A similar prevalence has even been reported in pediatric uremic patients [15] who presumably lack traditional atherosclerotic risk factors. The comorbidities in CKD patients that contribute to cardiovascular remodeling are atherosclerosis, hyperlipidemia, diabetes and/or hypertension, but also include a plethora of so-called non-traditional cardiovascular risk factors such as those discussed below and summarized in Table 1. These stimuli exacerbate the pathophysiological cardiac changes, including left-ventricular hypertrophy (LVH), diffuse interstitial fibrosis and capillary rarefaction leading to systolic and diastolic dysfunction. In this review, we consider these cardiac abnormalities that frequently occur in patients with CKD as uremic cardiomyopathy.

### 2.1. Left-Ventricular Hypertrophy in CKD

LVH is an independent predictor of cardiac death in dialysis patients. LVH can occur early in the course of CKD, even when the glomerular filtration rate (GFR) is still normal [16]. Once GFR is reduced, myocytes enlarge and cardiomyocytes expand, leading to LVH [17]. Cardiomyocytes are the major cell type, comprising about 70–85% of the total volume. They generate contractility by cyclic calcium fluxes [17,18]. Fibroblasts secrete collagen precursors and matrix metalloproteinases (MMPs) and thereby actively remodel the ECM [17,18,19]. The ECM embeds the myocytes and non-myocytes. Collagen is the most abundant protein here [19]. These structural changes are closely related to a functional impairment of the left ventricle causing diastolic dysfunction [20] while systolic function may, at least initially, remain normal [21]. LVH can be a consequence of increased preload due to hypervolemia or increased afterload due to increased peripheral resistance or hypertension, which are both very common in CKD. Further factors involved in the pathogenesis of LVH might be high cardiac output due to anemia or large arteriovenous fistulas for dialysis access [18,21].

### 2.2. Cardiac Fibrosis

In CKD patients, myocardial fibrosis is a pathologic process that occurs together with LVH. One hallmark of myocardial fibrosis is a marked increase in the production of extracellular matrix, especially collagens, which impair diastolic filling due to increased stiffness of the left ventricle, but they may also affect systolic function since functional myocytes are replaced by fibrotic scar tissue [22,23].

The main causes of myocardial fibrosis are hemodynamic alterations and disturbed secretion of various systemic soluble factors. A primary factor leading to myocardial fibrosis in CKD patients is hypertension, which is mainly associated with pressure overload. Factors related to hemodynamic alterations in CKD patients like senescence, ischemia, catecholamines, angiotensin II and aldosterone further promote the development of cardiac fibrosis [21]. Arterial stiffening is accelerated in the presence of CKD and is caused by a loss of elastic fibers and vascular calcification [24]. Increased vascular stiffness leads to increased cardiac afterload, which promotes cardiac hypertrophy and fibrosis [21,25]. 

However, during CKD progression, left ventricular remodeling has been found to occur even earlier than changes in large arteries [26]. This might be explained by a range of factors and mechanisms including uremic toxins, TGF-β and other growth factors. In addition to the role of hemodynamic changes in the development of cardiac fibrosis, non-hemodynamic factors related to the uremic milieu, such as overactivity of the renin-angiotensin-aldosterone system, FGF-23, parathyroid hormone, endothelin, increased sympathetic nerve discharge and increased plasma catecholamines might also play an important role [21,27]. 

Perivascular mesenchymal cells surround the VSMC layer in the so-called adventitia of larger arteries but are also present as pericytes around the vasa vasorum and in direct contact with endothelial cells of both the large arteries and micro-vessels. They play a role in vasomotion, homeostasis and permeability of the vasculature [11]. The heterogeneity of perivascular cells has been unclear for many years, but recent single-cell RNA-sequencing data suggest that various, previously unknown, perivascular mesenchymal populations exist [28,29]. We have reported that Gli1 marks a specific perivascular cell type that drives cardiac fibrosis and vascular calcification [30,31]. Gli1+ cells are a subset of cardiac interstitial PDGFRβ+ cells, but are mostly distinct from cardiac NG2+ pericytes [31]. However, the complex system of the cardiac perivascular cell types remains unclear, and single-cell experiments are needed to shed light on their heterogeneity and the role they play in homeostasis and disease. 

### 2.3. Capillary Rarefaction

Endothelial cells line blood or lymphatic vessels and are the most abundant cells of the non-myocyte fraction in murine hearts [6,7]. The medial layer of small and large vessels consists of vascular smooth muscle cells (VSMCs). Endothelial dysfunction is frequent in CKD [32] and can be considered an early manifestation of coronary vascular disease [33]. Endothelial dysfunction leads to disturbed microcirculation and is considered an independent risk factor for cardiovascular events [33,34]. CKD-induced microangiopathy has been shown to lead to tissue hypoxia and dysfunctional angiogenesis [33].

### 2.4. Oxidative Stress

Oxidative stress in CKD results from an imbalance in reactive oxygen species production and impaired antioxidant defense [35]. Various oxidation products have been shown to be overabundant in CKD. Increased ROS production has been reported to contribute to myocardial hypertrophy and fibrosis by lipid peroxidation, proinflammatory cytokines and DNA damage [35,36]. A marker of oxidative stress, 8-isoprostane, increases as CKD progresses [37]. Furthermore, NADPH oxidase generates reactive oxygen species, and this in turn leads to endothelial dysfunction [38]. Another effect of elevated oxidative stress is the oxidation of plasma proteins, which causes activation of phagocytes and increases inflammation [39]. Advanced oxidation protein products promote cardiomyocyte apoptosis. This process is mediated by upregulation of c-Jun N-terminal kinase (JNK) signaling and enhanced endoplasmic reticulum stress [40]. 

### 2.5. Inflammation

CKD can be seen as a state of increased systemic inflammation with various cytokines being among the recognized uremic toxins. The Chronic Renal Insufficiency Cohort Study (CRIC) study found that elevated plasma levels of high-sensitivity C-reactive protein (hs-CRP) and IL-6 were associated with LVH and systolic dysfunction in CKD patients [41]. Freise et al. stated that, amongst others, inflammatory processes involving tumor necrosis factor (TNF) and IL-10 impact pathobiological responses in arteries from children with CKD, and are thus associated with tissue remodeling and cardiovascular disease [42]. Furthermore, CKD patients develop endotoxemia, characterized by elevated levels of endotoxin, IL-6, CRP and lipopolysaccharide-binding protein (LBP), which contributes to chronic inflammation and has been associated with higher left-ventricular mass index (LVMI) and subsequently left-ventricular dysfunction [43]. Most of the soluble factors mentioned here are described as being secreted by cells of the heart (cardiomyocytes, endothelial cells, fibroblasts, VSMCs and pericytes) but also resident immune cells (e.g., macrophages, dendritic cells) and/or circulating cells might contribute to this. Thus, the contribution of inflammatory cells to the described mechanisms cannot be excluded.

### 2.6. Advanced Glycation end Products

The soluble receptor for advanced glycation end-products (sRAGE) seems to play an important role in cardiac remodeling in CKD patients. sRAGE has been described as a prognostic factor for mortality in diabetic dialysis patients [43,44]. Elevated sRAGE concentrations could represent a protective mechanism against the increased risk of cardiovascular complications resulting from AGEs and inflammation, although the underlying mechanisms need to be further confirmed [45]. It has also been shown that AGEs are involved in the upregulation of fibroblast growth factor 23 (FGF23).

In addition, cardiomyocytic FGF23 expression has been shown to be induced by activated renin- angiotensin aldosterone system (RAAS) [46]. In turn, FGF23-mediated activation of local RAAS in the heart promotes cardiac hypertrophy and fibrosis [47]. This finding underlines the organ crosstalk between the kidney and heart in CKD-CVD.

### 2.7. Growth Factors

In addition to the above-mentioned metabolic and inflammatory pathways, several growth factors might play important roles in cardiac fibrosis in CKD. One of the profibrotic modulators that stimulates fibroblast proliferation is FGF2. FGF2 binds to FGF-receptor (FGFR) 1, which is expressed in human cardiomyocytes. Additionally, FGF2 has been shown to promote growth of isolated cardiomyocytes [47,48] and cardiac hypertrophy in rats following myocardial infarction [49], thus further contributing to the cardiac phenotype in the cardiorenal syndrome. Another important growth factor-based mechanism of cardiac fibrosis development was found by analyzing human heart specimens. A marked reduction in cardiac Klotho, often found in CKD patients, was associated with increased TGF-β signaling. This in turn upregulated Wnt signaling, a major pathway in fibrosis. This was confirmed by in vitro studies with cardiomyocytes, where upregulation of endogenous Klotho inhibited Wnt/β-catenin signaling [50].

### 2.8. FGF23

FGF23 is a phosphaturic hormone primarily secreted by osteocytes. Its main actions are maintaining phosphate and mineral homeostasis. Furthermore, FGF23 decreases the synthesis of calcitriol. Its level rises early and dramatically with the decline of kidney function [51]. FGF23 has effects on various organs. Numerous studies have shown a correlation between serum FGF23 and cardiac alterations in CKD patients [52,53,54,55,56,57,58]. FGF23 directly induces LVH independent of preserved or reduced renal function; this has been shown in vitro in cultured cardiomyocytes and by in vivo studies in mice, as well as by correlations of circulating FGF23 levels with LVH in CKD patients [55]. FGF23 stimulates pro-fibrotic and pro-hypertrophic factors in cardiomyocytes and induces fibrosis-related pathways in fibroblasts [55,59,60]. In addition to its direct effects on cardiac remodeling, FGF23 has also been shown to increase blood pressure [61], inflammation [62] and CKD progression itself [63], and may thus promote the development of LVH also by indirect mechanisms. FGF23 signaling in the liver causes production of inflammatory cytokines. In the bone, FGF23 inhibits mineralization, leading to increased circulating phosphorus levels while reducing the production of erythropoietin (EPO) in the bone marrow [64]. All of the above-mentioned actions affect the outcome of CKD patients, and thus directly or indirectly lead to progression of cardiovascular disease. 

### 2.9. Klotho 

Membrane-bound Klotho serves as a co-receptor for FGF23 signaling and is synthesized in the kidney and bone. Soluble Klotho shows endocrine actions correlated with anti-aging effects [65]. Soluble Klotho levels are decreased in CKD [65]. Using Klotho-deficient mice, it could be shown that the Klotho-FGF23 axis plays a key role in pathologic cardiac remodeling in CKD, but also in phosphotoxicity and aging [66]. In pediatric CKD patients, serum FGF23 levels increased and Klotho levels decreased with progressing renal failure, while phosphorus levels were maintained in the normal range [67]. In those patients, high FGF23 and low Klotho levels were strongly associated with impaired left ventricular diastolic function [67]. In addition to this finding, FGF23 signaling via fibroblast growth factor receptor 4 (FGFR4) activates the phospholipase Cγ/calcineurin/nuclear factor of the activated T-cell pathway, and thus promotes cardiac myocyte hypertrophy, independent of its co-receptor Klotho [60,67]. Furthermore, FGFR4 signaling is responsible for FGF23-mediated increased cardiac contractility [68]. Correspondingly, aging mice lacking FGFR4 were protected from LVH. Thus, FGF23/FGFR4 signaling plays an important role in the regulation of cardiac remodeling and function [68]. Additionally, pharmacological blockade of the FGF receptor improved cardiac structure and function in 5/6 nephrectomy rats, thus underlining the role of FGFR activation as a mechanism of LVH in CKD [59].

### 2.10. Uremic Toxins

The role of uremic toxins in the development of cardiac remodeling is less well established. Recent work sheds light on the potential roles of asymmetric dimethylarginine (ADMA), advanced glycation end-products (AGE), trimethylamine N-oxide (TMAO) and indoxyl sulfate [69]. ADMA has been reported to be involved in regulation of nitric oxide, reactive oxygen species and renal anemia [69,70]. The AGE/RAGE axis is responsible for cell damage in CVD [69], driving cardiac fibrosis. TMAO, a metabolite derived from choline, is linked with left-ventricular diastolic dysfunction [71] and cardiac fibrosis [69]. 

In addition, several other uremic toxins are also thought to promote the cardiorenal syndrome by direct cardiotoxicity, including indoxyl sulfate and p-cresyl sulfate [71,72]. Indoxyl sulfate is a product of the intestinal catabolism of tryptophan with insufficient dialytic removal, as it is bound to albumin in the blood. In uremia, it increases up to 88-fold [73] and has been shown to mediate immune dysfunction and cytotoxic effects on endothelial cells in patients with end-stage renal disease [74]. Indoxyl sulfate stimulates the cannabinoid receptor type 1 and was associated with fibrotic effects via modulation of Akt signaling in myofibroblasts [75]. 

Several studies have pointed towards a role of uremic toxins in cardiac remodeling, however, the complex mechanisms of cell-type activation that drive cardiac fibrosis, hypertrophy and capillary loss are still unclear. Further studies using state-of-the-art single-cell technologies may shed more light on this important disease that affects numerous CKD/ESRD patients. 

## 3. Mouse Models of Cardiac Remodeling in CKD

To date, most animal studies investigating mechanisms and interventions in the cardiorenal syndrome have been performed in rats. The models employed (5/6 nephrectomy, adenine nephropathy, ischemia reperfusion injury, etc.) are well established and cause reliable cardiac remodeling. However, mouse models do offer some advantages, the most important of which is the possibility of generating genetic modifications, which enable the analysis of specific pathways and molecular mechanisms. Thus, improving mouse models of CKD-CVD is one important goal in order to clarify the pathogenesis of the cardiorenal syndrome and develop novel therapies for patients. Several attempts have been made to establish CKD-CVD in mice. They can be divided into three approaches: surgical interventions, chemical interventions and genetic interventions. Table 2 summarizes the kidney parameters and functional and structural cardiac parameters of murine CKD-CVD studies.

### 3.1. Surgically Induced Models

The most well-characterized surgical model is the subtotal nephrectomy model, also called 5/6 nephrectomy. There are different methods for performing this surgery; all involve unilateral nephrectomy. Reduction of the remaining kidney mass is then performed either by cauterization, ligation or slicing off the two poles, or by occlusion of the branches of the main kidney artery. Unilateral nephrectomy and reduction of the remaining kidney mass are either performed on the same day (1-step) or with a 7- to 14-day recovery phase after the unilateral nephrectomy (2-step). Various studies describe a cardiac phenotype resulting from CKD in these mice, which is aggravated with the time elapsed since surgery (see Table 2). The surgically induced model has been reported to mimic most phenotypic changes observed in the human disease. However, the resulting phenotype is highly dependent on the genetic background of the mice.

A study performed in nephrectomized C57BL/6 mice showed impaired renal function, anemia, cardiac hypertrophy, cardiac fibrosis and decreased systolic and diastolic heart function compared to sham-operated mice [75,89]. In addition, there was increased expression of natriuretic peptides, another marker of progressive heart failure, in nephrectomized male 129X1/SvJ mice [88]. Subtotal nephrectomy in male CD1 mice resulted in physiological and morphological changes that also mimicked the cardiac phenotype in patients with CKD [91]. In a study with male BALB/c mice, analyzed for up to 24 weeks after 5/6 nephrectomy, cardiac and arterial structure and function showed signs of fibrosis, oxidative damage and endothelial dysfunction [90,91]. 

RAAS is a key player in blood-pressure control and has been described as being highly relevant in the cardiorenal syndrome. Subtotal nephrectomy in angiotensin II type 1A receptor (AT1) knockout mice resulted in significantly reduced cardiac hypertrophy, fibrosis and capillary rarefaction compared to their wildtype littermates [87]. Other widely used cardioprotective and antihypertensive treatments such as β1-receptor blockers have also been shown to reduce cardiac hypertrophy in CKD [95].

CKD patients suffer from reduced erythropoietin (EPO) levels, and it has been shown that besides causing anemia, EPO reduction had direct effects on the cardiovascular system of these patients. In line with this, 5/6 nephrectomized mice receiving recombinant human erythropoietin (rhEPO) had a better outcome compared to saline-treated controls in terms of cardiac function and remodeling. These effects were independent of anemia. Thus, a control group that received an EPO derivate (asialo-EPO) still suffered from anemia but showed the same beneficial effects on cardiac remodeling [79,95]. Both compounds similarly attenuated LVH, indicating that EPO receptor signaling protected the hearts of CKD mice through mechanisms independent of erythropoiesis. The production of erythrocytes requires continuous stimulation of EPO receptors, whereas a brief stimulation is described to be sufficient for neuroprotection. The same mechanism might explain the cardioprotective effect in this model [79]. 

In addition, unilateral ureteral obstruction (UUO) can induce some cardiac hypertrophy and fibrosis [79,92]. However, since UUO does not cause renal failure in the presence of a non-injured contralateral kidney, the model is probably not suitable to induce a severe cardiac phenotype. 

By performing a graded ureter obstruction model in combination with a systemic expression of humanized S100A8, S100A12, and S100A9 in C57BL/6 mice, the association between S100/RAGE/FGF23 and cardiac hypertrophy was revealed [85]. Elevated serum concentrations of S100A12 are associated with inflammatory diseases, and thus might accelerate pathological cardiac remodeling in CKD patients. As mice do not express S100A12 [34,96], a humanized model was generated and CKD was induced via reversible unilateral ureteral obstruction. For this method, the right ureter was obstructed using a clip that was relocated every other day to prevent irreversible obstruction. After 7 days, the clip was removed to allow recovery of the right kidney, followed by irreversible ligation of the left ureter. A potential mechanism to explain the results of the study was presented with the finding that in addition to osteocyte expression, in CKD patients, FGF23 is also expressed in the heart by cardiomyocytes, cardiac fibroblasts, vascular smooth muscle cells and endothelial cells in coronary arteries, and by inflammatory macrophages [60]. It was suggested that S100/RAGE-mediated chronic sustained systemic inflammation caused cardiac fibroblasts to upregulate FGF23 synthesis, and in turn, increased cardiac FGF23 levels were linked to pathological cardiac remodeling [86]. 

### 3.2. Chemically Induced Models

Surgical methods to induce CKD associated with cardiovascular changes are often accompanied by a high mortality and require well-trained surgeons. Thus, efforts have been made to establish non-surgical techniques for CKD induction. One option is to apply orally administered adenine, which is metabolized to 2,8-dihydroxyadenine, and which in turn precipitates as crystals in the renal proximal tubular epithelium causing inflammation and fibrosis, ultimately leading to CKD. Indeed, mice treated with adenine developed symptoms of the cardiorenal syndrome, in particular cardiac hypertrophy, impaired cardiac function, as well as increased fibrosis [97].

An additional chemically induced model for murine CKD-CVD was achieved in mice via a single injection of cisplatin and subsequent feeding of a high-phosphate diet. CKD was confirmed by decreased creatinine clearance, development of interstitial kidney fibrosis, hyperphosphatemia, high plasma levels of PTH and FGF23 and low levels of plasma calcitriol and αKlotho. The mice developed LVH and cardiac fibrosis. This model resembles the transition from acute kidney injury to chronic renal failure and thus displays a promising approach to study underlying mechanisms in humans [93]. 

### 3.3. Genetically Induced Models

In addition to the above-mentioned models for CKD induction, CVD has also been reported in genetic mouse models of CKD. One example is the Alport mouse model, i.e., murine Col4a3 deficiency. The phenotype of C57BL/6 Col4a3 knockout mice was milder than that of 129Sv mice, which correlated with prolonged survival of the C57BL/6 mice. After 20 weeks, the C57BL/6 mice developed CKD associated with functional and structural symptoms of cardiac remodeling [94]. This emphasizes the importance of the genetic background of the mice used in relation to the severity of cardiac disease. For example, C57BL/6 mice usually have much milder phenotypes compared to 129Sv mice. 

## 4. Potential Therapeutic Targets of Cardiac Remodeling in CKD

Reduction or inhibition of LVH might be achieved by non-specific treatments such as reduction of hypervolemia, lowering of blood pressure and treatment with angiotensin-converting enzyme inhibitors or angiotensin receptor blockers that exhibit potential direct effects on the myocardium [16,94]. Further strategies to prevent left-ventricular remodeling in patients with mild-to-moderate CKD comprise of reducing overweightness and avoiding hemoglobin concentrations that are too high [98]. 

Paricalcitol, a vitamin D receptor agonist, has shown a beneficial effect on myocardial fibrosis in rats [77,78]. However, in CKD patients, paricalcitol administration failed to improve diastolic function [99,100]. 

As oxidative stress triggers cardiovascular remodeling, antioxidative therapies have been proposed to protect endothelial cells from reactive oxygen species, thereby preventing endothelial dysfunction. Quercetin and antioxidant enzyme mimetics were shown to inhibit the NFkB pathway and reduce ROS generation in an in vitro assay using endothelial cells [101]. Several studies have attempted to reduce imbalances caused by oxidative stress in cardiac remodeling in CKD. Accordingly, Liu et al. demonstrated that antagonism of the Na/K-ATPase ameliorates uremic cardiomyopathy in 5/6 nephrectomized mice [81,82]. Na/K-ATPase activation leads to increased ROS production and acts as a signal transducer inside cardiomyocytes [81]. In activated Na/K-ATPase signaling, microRNA 29b-3b is downregulated, and thus fails to inhibit collagen expression, which has been shown in cardiac fibroblasts [81,83]. Na/K-ATPase is also known to stimulate mTOR signaling, thereby activating pro-fibrotic pathways. Moreover, the mTOR inhibitor rapamycin has been shown to inhibit cardiac fibrosis in rats [102]. 

As some of the uremic toxins are derived from intestinal bacteria, the microbiome could be another potential target in the cardiorenal syndrome. Thus, indoxyl sulfate and para-Cresyl sulfate serum levels were reduced by antibiotic therapy in ESRD patients [103,104]. Indoxyl sulfate binds to the cannabinoid receptor [75]. Interestingly, inhibition of this receptor has also been shown to reduce cardiac fibrosis in 5/6 nephrectomized mice [75]. 

FGF23 promotes cardiac fibrosis and LVH. Additionally, FGF23 signaling in the liver causes the production of inflammatory cytokines. In the bone, FGF23 inhibits mineralization, leading to increased circulating phosphorus levels and a reduction in the production of EPO [64,75]. Thus, the identification of FGF23 receptors in the respective organs will be crucial in future research since their selective blockade could be considered as a therapeutic target [64]. On the other hand, FGF23 antibody treatment caused mineral disturbances, in particular hyperphosphatemia and was associated with increased mortality in a CKD rat model [105]. Recombinant Klotho was used in an experimental mouse model to attenuate cardiac remodeling and reduce cardiac and renal fibrosis [105,106]. A positive correlation between FGF23 and cardiac hypertrophy exists in a Klotho-deficient state, but not in a Klotho-repleted state [76,105,106]. In addition, pharmacological interference with cardiac FGF23/FGFR4 signaling might have a protective effect on CKD- and age-related LVH [68]. First studies in rats report that pharmacological inhibition of FGFR might be a potent blood pressure-independent mechanism to prevent LVH in CKD [59].

CKD patients have low EPO levels, which are associated with cardiac fibrosis. A study in 5/6 nephrectomized rats showed that EPO in combination with enalapril reduced cardiac fibrosis and capillary rarefaction. The underlying mechanisms are likely multifactorial but may encompass decreased myocardial oxidative stress [81]. In CKD patients, there is better survival in those treated with EPO to a hemoglobin level of 10-12 g/dl, whereas normalization of hemoglobin levels was not beneficial [80,107]. 

Calò et al. identified rho kinase (ROCK) activation as a potential LVH marker in CKD patients, which indicates that inhibition of ROCK activation might serve as a target to treat cardiac remodeling in those patients [108].

A study performed in 5/6 nephrectomized rats treated with the PPARα agonist clofibrate did indeed reveal improved cardiac function and prevention of LVH [84]. 

Finally, restoring kidney function can at least partially reverse cardiac changes. It has been shown by echocardiography that a kidney transplantation improves left-ventricular function [103]. 

In conclusion, to date, few specific therapies exist that can inhibit cardiac remodeling in CKD. A better understanding of the cell types involved and their mechanisms of activation, e.g., by crosstalk or presumed uremic toxins, will guide the development of urgently needed novel therapeutics. 

## Figures and Tables

**Table 1:** Some systemic factors that have been reported to be involved in uremic cardiomyopathy.

	Factor	References
**Phosphate Homeostasis**		
	FGF23/Klotho	[46,47,50,52,53,54,55,56,57,58,59,60,67,68,76]
	Vit D receptor agonists	[77,78]
**Uremic Toxins**		
	p-Cresylsulfate	[72]
	Indoxylsulfate	[72,75]
	ADMA	[69,70]
**Growth Factors**		
	TGF-β	[50]
	FGF2	[48,49]
	EPO	[64,79,80]
**Metabolic Stress**		
	AGE	[44,45,69]
	ROS	[36,38,40,81,82,83]
	PPARα	[84]
	TMAO	[69,71]
**Inflammation**		
	S100/calgranulin	[85,86]
	Interleukin 6	[43]
	Interleukin 10	[42]
	CRP	[41]
	TNF	[42]

**Table 2:** Functional and structural cardiac parameters in mouse models of chronic kidney disease (CKD). GFR—glomerular filtration rate, sCr—serum creatinine, BUN—blood urea nitrogen, EF—ejection fraction, FS—fractional shortening, SV—stroke volume, CO—cardiac output, BP—blood pressure, LVH—left-ventricular hypertrophy, Nx—nephrectomy, AT1—angiotensin II type-1A receptor, UUO—unilateral ureteral obstruction, hBAC-S100—bacterial artificial chromosome of the human S100/calgranulin gene cluster, Col4a3—collagen type IV alpha 3 chain, n.d.—not determined, wk—week.

.			Kidney			Heart (Functional)			Heart (Structural)			
Model	Mouse Strain	Duration (Weeks)	GFR	sCr	BUN	EF/FS	SV/CO	BP	LVH	Fibrosis	Capillary Loss	References
*surgically induced*												
5/6 Nx (2-step)	C57BL/6	8	n.d.	↑	↑	-	↓	n.d.	↑	↑	n.d.	[75]
5/6 Nx (2-step)	C57BL/6	12	n.d.	↑	↑	↓	n.d.	n.d.	↑	↑	↑	[87]
AT1 knockout, 5/6 Nx (2-step)	C57BL/7	12	n.d.	↑	↑	↓	n.d.	n.d.	↑	↑	-	[87]
5/6 Nx (2-step)	C57BL/6	12	n.d.	↑	↑	↓	n.d.	-	↑	↑	↑	[79]
5/6 Nx (2-step)	129X1/SvJ	16	n.d.	n.d.	↑	-	n.d.	↑	↑	↑	n.d.	[88]
5/6 Nx (2-step, pole ligation)	C57BL/6	4	n.d.	↑	↑	n.d.	n.d.	n.d.	↑	↑	n.d.	[89]
5/6Nx (1-step)	BALB/c	8, 16 and 24	n.d.	n.d.	↑ (8, 16, 24 wk)	↓ (24 wk)	n.d.	↑ (16, 24 wk)	n.d.	↑ (24 wk)	n.d.	[90]
5/6Nx (1-step)	CD1	4, 6 and 8	n.d.	n.d.	n.d.	↑	n.d.	↑	↑	↑	n.d.	[91]
UUO	C57BL/6	3	n.d.	↑	↑	-	n.d.	↑	↑	↑	-	[92]
hBAC-S100, UO	C57BL/6	10	n.d.	n.d.	↑	-	n.d.	↑	↑	n.d.	n.d.	[85]
	129Sv	10	↓	↑	↑	↓	↓	↑	-	↑	n.d.	
*chemically induced*												
0.15% adenine	C57BL/6	20	↓	↑	↑	↓	n.d.	n.d.	↑	↑	n.d.	[86]
10 mg/kg cisplatin + high phosphate diet	129Sv	20	↓	↑	↑	n.d.	n.d.	n.d.	↑	↑	n.d.	[93]
*genetically induced*												
Col4a3 knockout	C57BL/6	10 and 20	↓	↑	↑	-	↓ (only 20 wk)	↑ (only 10 wk)	↑ (only 20 wk)	↑ (only 20 wk)	n.d.	[94]

↓: reduced in comparison to control animals ↑: increased in comparison to control animals.
